# Rbm24 displays dynamic functions required for myogenic differentiation during muscle regeneration

**DOI:** 10.1038/s41598-021-88563-3

**Published:** 2021-05-03

**Authors:** Raphaëlle Grifone, Audrey Saquet, Manon Desgres, Claudia Sangiorgi, Caterina Gargano, Zhenlin Li, Dario Coletti, De-Li Shi

**Affiliations:** 1 Laboratory of Developmental Biology (LBD), CNRS UMR7622, Institut de Biologie Paris-Seine (IBPS), Sorbonne Université, 75005 Paris, France; 2 Biological Adaptation and Ageing (B2A), CNRS UMR8256 and INSERM U1164, Institut de Biologie Paris-Seine (IBPS), Sorbonne Université, 75005 Paris, France; 3 Department of Anatomy, Histology, Forensic Medicine and Orthopedics, Histology and Medical Embryology Section, Sapienza University of Rome, 00161 Rome, Italy

**Keywords:** Muscle stem cells, Stem-cell differentiation, Cell biology, Stem cells

## Abstract

Skeletal muscle has a remarkable capacity of regeneration after injury, but the regulatory network underlying this repair process remains elusive. RNA-binding proteins play key roles in the post-transcriptional regulation of gene expression and the maintenance of tissue homeostasis and plasticity. Rbm24 regulates myogenic differentiation during early development, but its implication in adult muscle is poorly understood. Here we show that it exerts multiple functions in muscle regeneration. Consistent with its dynamic subcellular localization during embryonic muscle development, Rbm24 also displays cytoplasm to nucleus translocation during C2C12 myoblast differentiation. In adult mice, *Rbm24* mRNA is enriched in slow-twitch muscles along with *myogenin* mRNA. The protein displays nuclear localization in both slow and fast myofibers. Upon injury, Rbm24 is rapidly upregulated in regenerating myofibers and accumulates in the myonucleus of nascent myofibers. Through satellite cell transplantation, we demonstrate that Rbm24 functions sequentially to regulate myogenic differentiation and muscle regeneration. It is required for *myogenin* expression at early stages of muscle injury and for muscle-specific pre-mRNA alternative splicing at late stages of regeneration. These results identify Rbm24 as a multifaceted regulator of myoblast differentiation. They provide insights into the molecular pathway orchestrating the expression of myogenic factors and muscle functional proteins during regeneration.

## Introduction

Skeletal muscle, the most abundant tissue in vertebrates, is composed of highly specialized post-mitotic cells that contract to generate force and movement required for locomotor activity, postural behavior and breathing. While this tissue is susceptible to get injured after direct trauma or under genetic disorders, it has the remarkable ability to self-repair by orchestrating fine-tuned cellular responses, resulting in a totally functional muscular apparatus^1–3^. The process of muscle regeneration recapitulates embryonic skeletal muscle development, during which embryonic muscle genes are re-expressed and central nuclei are visible in regenerating fibers^4–6^. This powerful regenerative capacity mostly relies on the expansion, differentiation and maturation of a resident pool of quiescent muscle stem cells, termed satellite cells, owing to their location between the plasma membrane of each myofiber, namely the sarcolemma, and its surrounding basal lamina^7,8^. Their location within this unique niche in intact muscle maintains them in a mitotically dormant, quiescent state^7,8^. In addition to their specific location, quiescent satellite cells can be identified within the muscle tissue by the expression of several markers, among which Pax7 is considered as the main defining factor for this cell type^9–11^.

Muscle regeneration involves different cellular behaviors and regulatory networks that function at each stage of the repair process^3^. Upon stimulation, such as muscle damage, intense exercise, or pathogenic conditions, all providing a local burst of extracellular signals, Pax7-positive quiescent satellite cells are activated to re-enter the cell cycle and undergo proliferation. They subsequently express myogenic regulatory factors (MRFs), namely MyoD, Myf5, myogenin, and MRF4, as well as other genes, such as the embryonic isoform of myosin, and differentiate to fuse with each other or with existing myofibers to repair the injured muscle tissue^12–14^. A rapid response to this variety of stimuli to drive the differentiation of satellite stem cells is partly ensured by the regulation of mRNA stability, allowing a rapid increase in the level and availability of regulatory factors. Consistently, RNA-binding proteins (RBPs) regulating mRNA stabilization have been shown to be preferentially upregulated in satellite cells following muscle injury^15^. Further supporting this observation, there is accumulating evidence that RBPs are necessary for normal muscle physiology. In particular, several RBPs, including HuR, FXR1P, Rbm20, Rbm24, Rbm38, and CELF, have been shown to promote muscle cell differentiation by stabilizing mRNAs encoding MRFs and/or by regulating alternative splicing of muscle differentiation genes^16–21^. Other RBPs, such as AUF1, ZFP36, and KSRP, have been also shown to bind to AU-rich elements of specific target mRNAs and modulate the expression of various MRFs that function at different phases of myogenesis to ensure normal skeletal muscle development^22^. In further support of their pivotal role in muscle homeostasis, mutations or dysfunctions of RBPs have been shown to cause skeletal muscle-related diseases in humans, such as myotonic dystrophy, facioscapulohumeral muscular dystrophy, and oculopharyngeal muscular dystrophy^16,23,24^. These observations clearly point out RBPs as critical players in the post-transcriptional regulation of gene expression to promote myogenic differentiation.

Rbm24 (RNA binding motif protein 24), one of these post-transcriptional regulators of embryonic lineage differentiation and tissue homeostasis, contains a single RNA recognition motif (RRM) at the N-terminal region and an uncharacterized C-terminal region^25^. Several studies have reported an essential role for Rbm24 during myogenesis and somitogenesis in vertebrate embryos as well as in cultured cell lines^26–30^. However, the molecular mechanism by which Rbm24 post-transcriptionally regulates muscle differentiation has just begun to be elucidated. On the one hand, altered alternative splicing of muscle structural and functional genes has been observed following loss of Rbm24 in myoblasts^21,29^. On the other hand, there is compelling evidence that it is involved in modulating mRNA stability and translation in myoblast cell lines^17,31^. Its implication in regulating different post-transcriptional events that are spatially compartmentalized suggests that it may have dynamic subcellular localization and function during muscle development. Indeed, we have shown that Rbm24 protein was distributed in the cytoplasm of MyoD-positive myoblasts within the myotome and required for early steps of myogenic differentiation in vertebrate embryos^28^. However, its expression and regulatory roles in differentiated myofibers, particularly in adult muscle, remain open for further investigation.

In the present study, we analyzed the subcellular localization and function of Rbm24 in mouse adult muscle and myoblast cell line. Our results showed that Rbm24 protein is first retained in the cytoplasm of myoblasts and then translocated to the nucleus of myotubes and myofibers. This nuclear localization of Rbm24 in adult skeletal muscles implies that it may play a role in muscular function and/or muscle regeneration. Using a mouse muscle injury model induced by cardiotoxin (CTX), we demonstrated that Rbm24 plays a role in skeletal muscle regeneration from satellite cells through regulation of *myogenin* mRNA expression at early stages of muscle injury and alternative splicing of muscle-specific genes at late stages of regeneration. Altogether, these findings reveal a dynamic function of Rbm24 in the process of muscle repair, which could rely on a fine-tuned regulation of its subcellular localization and post-transcriptional activity during the differentiation of satellite cells.

## Results

### Rbm24 is enriched in adult slow muscles and restricted to the myonucleus

**Figure 1:**
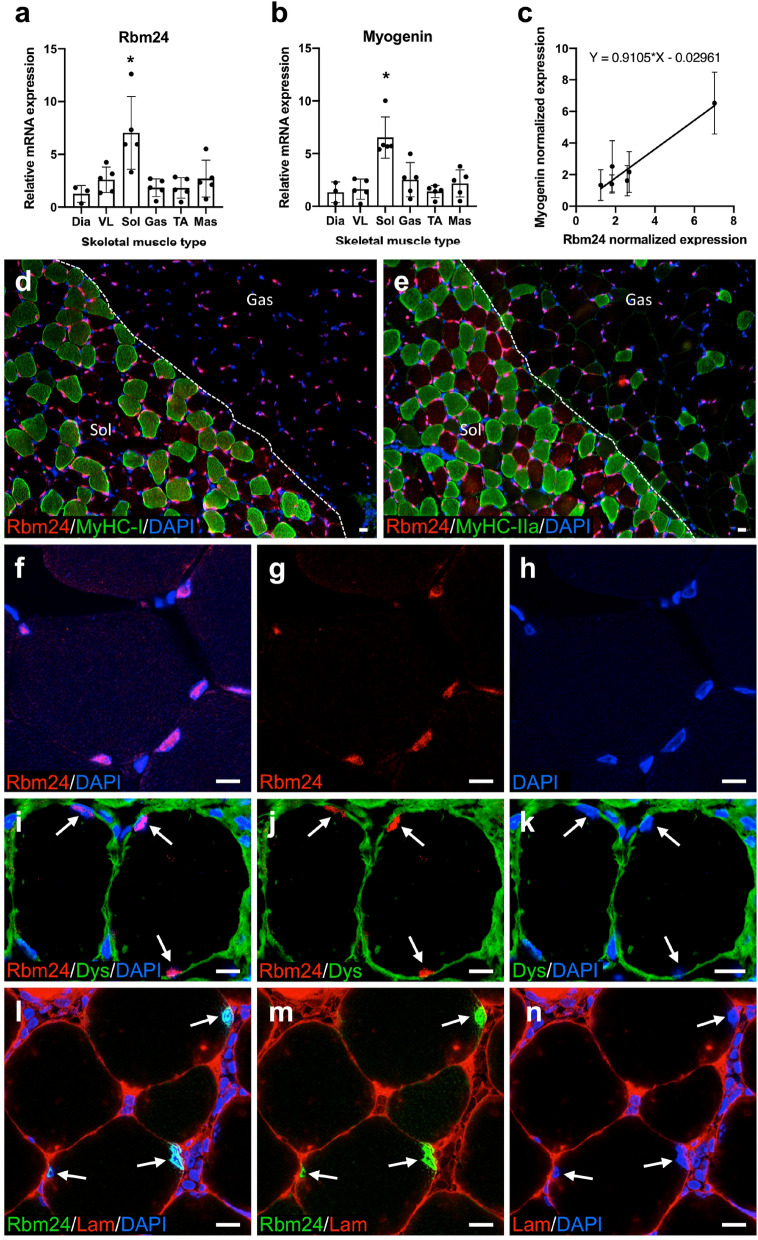
Rbm24 expression in mouse adult muscles. (**a**,**b**) Analyses by qRT-PCR of *Rbm24* (**a**) and *myogenin* (**b**) expression in different skeletal muscle types. *Dia* diaphragm, *VL* vastus lateralis, *Sol* soleus, *Gas* gastrocnemius, *TA* tibialis anterior, *Mas* masseter. The expression level of *Rbm24* or *myogenin* in the diaphragm is set to 1 as a reference. Data are the mean ± s.e.m. from three independent experiments. ANOVA F (df 5.22) = 6.333, *p* < 0.001 for *Rbm24*; ANOVA F (df 5.22) = 10.55, *p* < 0.0001 for *myogenin*; *p < 0.05 by Tukey’s HSD test. Note the higher levels of *Rbm24* and *myogenin* expression in the slow-twitch soleus muscle compared to the five other muscles enriched in fast-twitch myofibers. (**c**) Linear correlation between *Rbm24* and *myogenin* expression in different muscles. R^2^ = 0.6527. (**d**,**e**) Double immunostaining on cryosections of soleus (Sol) and gastrocnemius (Gas) muscles shows Rbm24 nuclear localization in type I and type IIa myofibers, as well as in other fiber types devoid of MyHC-I or MyHC-IIa staining in the gastrocnemius. Note that more intense Rbm24 nuclear staining is present in the soleus muscle. (**f**–**h**) Immunolocalization of Rbm24 in the myonucleus of tibialis anterior muscle, as visualized by counterstaining with DAPI. (**i**–**n**) Double immunofluorescence staining of Rbm24 and dystrophin (Dys) or laminin (Lam) on cryosections of tibialis anterior muscle shows the localization of Rbm24 protein at the periphery of myofibers, inside the dystrophin- and laminin-associated muscle cell membrane (arrows). Note the absence of Rbm24 staining in nuclei outside the sarcolemma. Scale bars: 10 µm.

Rbm24 displays a highly conserved expression pattern during vertebrate early development^28,32^, and is mostly expressed in skeletal and cardiac muscles in the adult^21^. Because muscle regulatory factors may be differentially involved in the differentiation and function of fast versus slow muscles^33^, the relative expression of *Rbm24* gene in different muscle types merits further investigation. To clarify this issue, we used 12-week-old mice to examine *Rbm24* expression in six different skeletal muscles that vary in fiber type composition. By qRT-PCR analysis, we found that the expression level of *Rbm24* in the soleus muscle, which is enriched in slow-twitch myofibers, was four times higher than that in the five other skeletal muscles with fast or mixed fiber types, including masseter, vastus lateralis, gastrocnemius, diaphragm, and tibialis anterior (Fig. 1a). It has been shown that *myogenin* mRNA is a target of Rbm24 protein^31^, and is preferentially expressed in the soleus muscle^34–36^. Our qRT-PCR analysis not only confirmed a high level of *myogenin* expression in the soleus muscle (Fig. 1b), but also showed a linear correlation in the expression of *Rbm24* and *myogenin* in different skeletal muscles (Fig. 1c). Thus, these results reveal a specific enrichment of *Rbm24* mRNA in the slow muscle and raise the possibility that it may play a role in regulating *myogenin* expression and the specification of adult muscle fiber types.

Our previous study showed that Rbm24 protein was accumulated in the cytoplasm of MyoD-positive myoblasts within the myotome, but not in Pax3-positive premyogenic progenitors^28^. To examine the subcellular localization of Rbm24 protein in adult muscle, we first performed double immunofluorescence staining on cryosections of slow soleus and fast gastrocnemius muscles using an Rbm24-specific antibody as reported in our previous studies^28,32^, along with muscle-specific myosin heavy chain (MyHC) isoforms, MyHC-I and MyHC-IIa. This showed a nuclear localization of Rbm24 in the periphery of the two muscle fiber types composing the slow soleus muscle and expressing type I and IIa myosin heavy chain. This also revealed a nuclear accumulation in the periphery of the MyHC-I and MyHC-IIa negative thus MyHC-IIb and MyH-IIx expressing myofibers composing the fast gastrocnemius muscle. Consistent with the observation from qRT-PCR analysis, Rbm24 protein seems to be slightly enriched in nuclei of the slow soleus muscle fibers compared to nuclei of the adjacent fast gastrocnemius on the same section (Fig. 1d,e). We then performed immunodetection on cryosections of the tibialis anterior to further precise the location of Rbm24-positive nuclei in the muscle. The results clearly showed that Rbm24 was only accumulated in some nuclei at the periphery of myofibers, as evidenced by DAPI staining (Fig. 1f–h). Furthermore, double immunofluorescence staining of Rbm24 with either dystrophin (Fig. 1i–k) or laminin (Fig. 1l–n) unequivocally showed that Rbm24-positive nuclei were exclusively positioned within the myofiber, inside the dystrophin- and laminin-associated muscle cell membrane, but not outside the sarcolemma. These results clearly demonstrate that the localization of Rbm24 protein in adult muscle is restricted to myofiber nuclei, but is excluded from all outer mononucleated cells, among which are satellite cells that localize between the sarcolemma and the basal lamina.

### Rbm24 shuttles from the cytoplasm to the nucleus during myogenic differentiation

**Figure 2:**
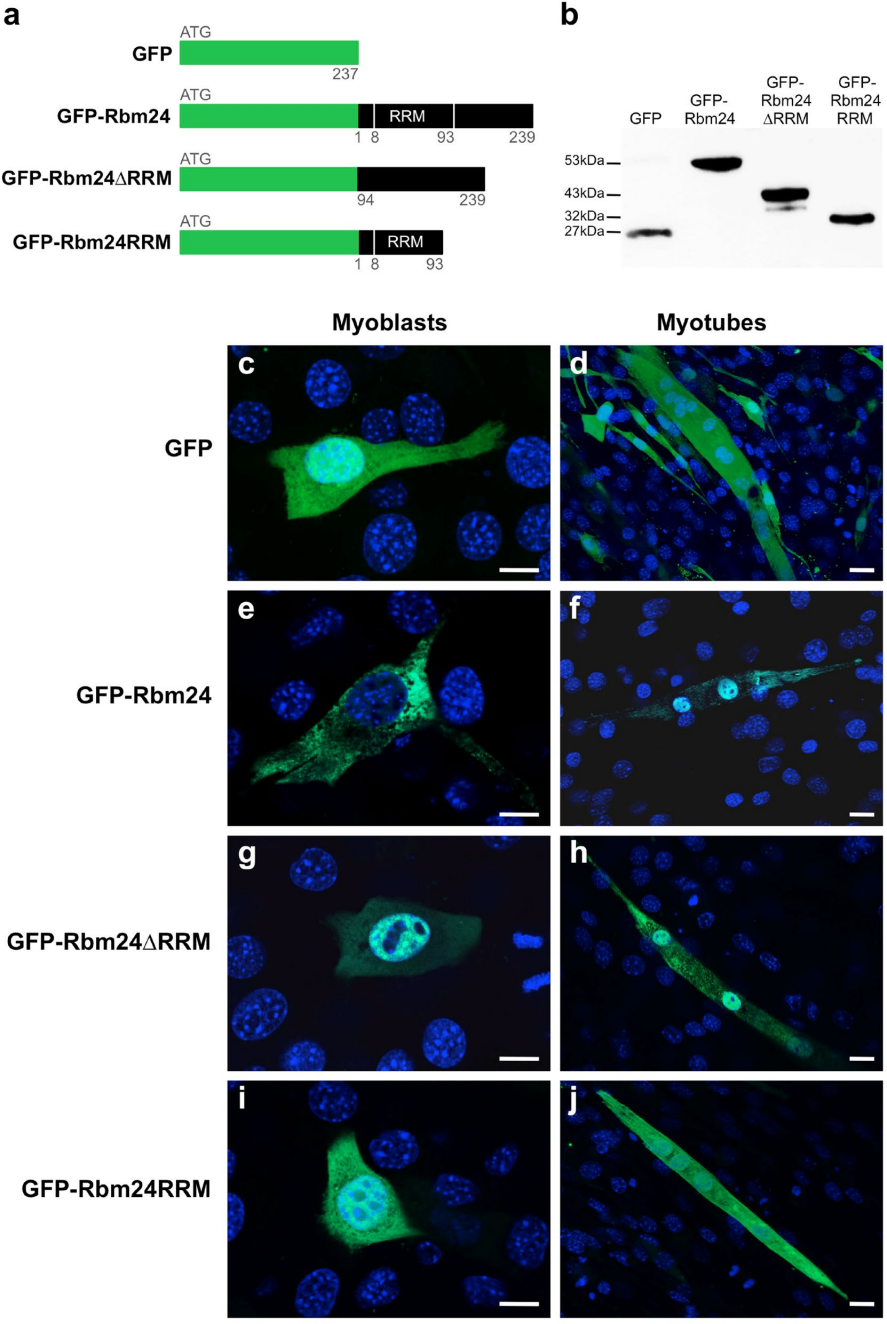
Cytoplasm to nucleus translocation of Rbm24 during myogenic differentiation in C2C12 cells. (**a**) Schematic representation of GFP-tagged full-length and truncated forms of Rbm24 proteins. Numbers indicate amino acid residues in mouse Rbm24 protein. (**b**) Western blot analysis of indicated fusion proteins in C2C12 myoblasts 24 h after transfection using a GFP antibody. Full-length blot is presented in Supplementary Fig. S1. (**c**–**j**) Subcellular localization of different fusion proteins after transient transfection in C2C12 cells, which were counterstained with DAPI. (**c**,**d**) GFP alone was uniformly distributed in proliferating myoblasts 24 h after transfection and in multinucleated myotubes after 3 days of differentiation. (**e**,**f**) GFP-Rbm24 was heterogeneously distributed in the cytoplasm of myoblasts, but was predominantly localized in the nucleus of differentiated myotubes. (**g**,**h**) GFP-Rbm24ΔRRM was mainly localized in the nucleus of myoblasts, and was distributed both in the cytoplasm and nucleus of differentiated myotubes. (**i**,**j**) GFP-Rbm24RRM was uniformly distributed in proliferating myoblasts and in multinucleated myotubes. Scale bars: 10 µm.

We previously showed that Rbm24 was accumulated in the cytoplasm of fate-committed myoblasts^28^. The nuclear localization of Rbm24 in mature adult myofibers observed in the present work suggests that there is a dynamic trafficking of this protein during myogenic differentiation. This was further confirmed by following the localization of GFP-tagged full-length Rbm24 (GFP-Rbm24) and its truncated forms lacking either the N-terminal RRM (GFP-Rbm24∆RRM) or the C-terminal half (GFP-Rbm24RRM) during differentiation of C2C12 myoblasts (Fig. 2a). Western blot analysis showed that these fusion proteins were correctly produced in C2C12 cells transfected with the corresponding plasmids (Fig. 2b). Their subcellular distribution was examined 24 h after transfection, at which time C2C12 cells were in a proliferative myoblast state, and after 3 days of differentiation induced by low serum, when myoblasts fused into multinucleated myotubes. While GFP alone was uniformly distributed in the cytoplasm and the nucleus of myoblasts and myotubes (Fig. 2c,d), GFP-Rbm24 was heterogeneously localized in the cytoplasm but not in the nucleus of myoblasts (Fig. 2e), which was similar as observed by immunostaining of Rbm24 protein in fate-committed myoblasts during embryogenesis^28^. Upon differentiation, however, Rbm24 was mostly translocated to the nucleus of myotubes (Fig. 2f). Interestingly, GFP-Rbm24∆RRM harboring only the C-terminal region displayed preferential localization in the nucleus of both myoblasts and myotubes (Fig. 2g,h). This implies that the RRM plays a role to maintain Rbm24 in the cytoplasmic compartment, thus allowing the protein to stabilize its targets, such as *myogenin, p21*, and *p63* mRNAs, as shown in C2C12 myoblasts or in other cell lines^31,37,38^. However, GFP-Rbm24RRM lacking the C-terminal region showed a uniform cellular accumulation in myoblasts and myotubes (Fig. 2i,j), suggesting that the RRM alone is not sufficient for the cytoplasmic localization, which should be assisted by the C-terminal region.

Altogether, these results reveal that Rbm24 displays dynamic subcellular localization during myogenic differentiation of skeletal muscle cells. The protein resides first in the cytoplasm of myoblasts, where it could function to stabilize target mRNAs involved in the early differentiation steps^31^, and then translocates to the nucleus of myotubes and myofibers to regulate muscle-specific alternative splicing of target genes related to late steps of muscle differentiation and muscular function^21,29^. Thus, Rbm24 should be implicated in multiple aspects of post-transcriptional regulation underlying skeletal muscle development.

### Rbm24 expression is increased in regenerating skeletal muscle

**Figure 3:**
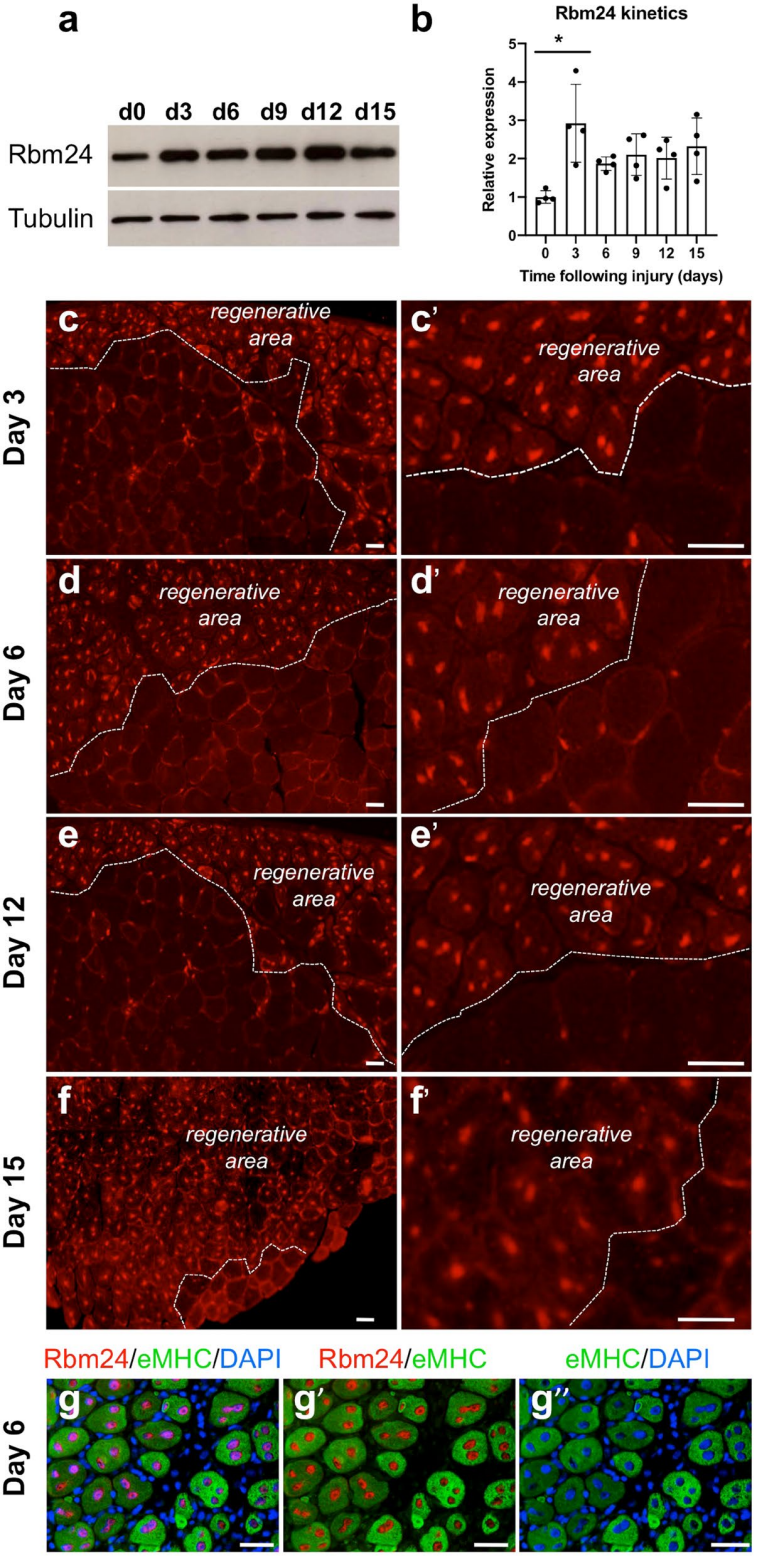
Rbm24 expression during skeletal muscle regeneration in mice. (**a**) Western blot analysis of Rbm24 protein in the tibialis anterior muscle of adult mice at 3, 6, 9, 12 and 15 days of regeneration. Control muscle was the contralateral tibialis anterior injected with PBS. Tubulin was used as a loading control. Full-length blots are presented in Supplementary Fig. S2. (**b**) Quantification of Rbm24 protein levels followed by ANOVA analysis shows increased expression of Rbm24 after muscle injury. Rbm24 protein level in control muscle harvested at day 0 is set to 1 as a reference, after normalization with tubulin. Data are the mean ± s.e.m. from three independent experiments. ANOVA F (df 5.18) = 4.247, p < 0.01; *p < 0.05 by Tukey’s HSD test. (**c**–**f′**) Immunofluorescence staining on cryosections of mouse adult tibialis anterior muscle at 3, 6, 12 and 15 days of regeneration shows increased expression of Rbm24 in the nucleus of regenerating myofibers. For all time points, low (left panel) and higher (right panel) magnifications are shown. White dotted lines delimit regenerating areas composed of newly formed myofibers with centralized nuclei and uninjured myofibers with peripheral nuclei. (**g**–**g″**) Double immunofluorescence staining of Rbm24 and eMHC proteins in the tibialis anterior at 6 days of regeneration. As the first myosin isoform expressed in developing muscle fibers, eMHC is re-expressed at early stages of muscle regeneration. DAPI was used to stain nuclei. Rbm24 protein is localized in centralized nuclei of newly formed myofibers that repair the injured muscle tissue, but not in nuclei outside the myofibers. Scale bars: 10 µm.

Given the importance of Rbm24 in myogenic differentiation during embryonic development, its specific localization in the myonucleus led us to explore the possibility whether it may participate in skeletal muscle regeneration as well. For this purpose, we first analyzed its temporal expression in the tibialis anterior following CTX-induced injury, because of the accessibility of this muscle for manipulation. In comparison with uninjured muscle, western blot analysis showed a two to three fold increase in Rbm24 protein level 3 days after injury, which was maintained until at least 15 days of regeneration (Fig. 3a,b). Next, we examined Rbm24 localization on cryosections of regenerating muscles. Immunofluorescence staining indicated that Rbm24 was strongly expressed in regenerating myofibers at different time points when compared to uninjured muscles on the same section (Fig. 3c–f). Higher magnification images showed that Rbm24 protein was predominantly localized to centralized myonuclei within the regenerating zone from 3 days of regeneration onward (Fig. 3c′–f′). In addition, double immunofluorescence staining of Rbm24 and embryonic myosin heavy chain (eMHC), a protein known to be transiently upregulated in immature regenerating myofibers but downregulated in matured myofibers, further confirmed the specific nuclear localization of Rbm24 in eMHC-positive newly formed myofibers at 6 days of regeneration (Fig. 3g–g″).

It was not possible for us to determine Rbm24 localization in proliferating myoblasts at more early stages of regeneration, for example, at day 1 or day 2 after injury, due to the presence of various inflammatory cell populations in the severely damaged tissue, such as monocytes and macrophages that interfere with immunofluorescence staining. The use of Rbm24 transgenic in future works may help to circumvent this difficulty. Nevertheless, Rbm24 expression was strongly expressed in early newly formed myofibers, which are formed from muscle satellite cells^39^. Thus, our results suggest that *Rbm24* gene becomes re-activated in these stem cells when they re-enter the cell cycle to proliferate and differentiate into myoblasts. As observed in C2C12 cells, it is possible that in the context of muscle regeneration, endogenous Rbm24 protein may be first localized in the cytoplasm of mononucleated myoblasts derived from satellite cells, and then accumulated in multinucleated myofibers.

### Experimental set-up to interfere with Rbm24 functions during skeletal muscle regeneration

**Figure 4:**
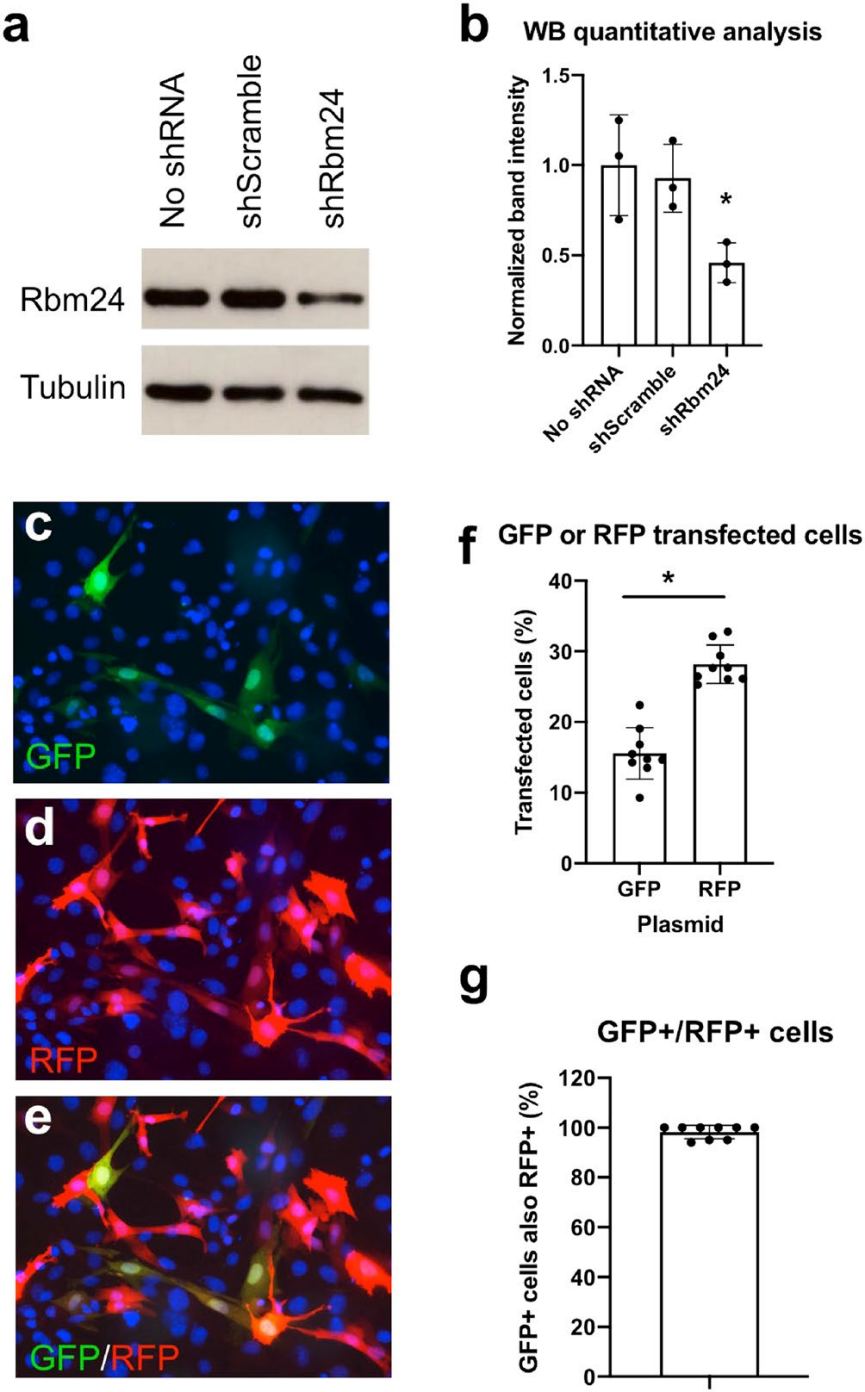
Experimental set-up for the analysis of Rbm24 functions in muscle regeneration. (**a**) Western blot analysis of Rbm24 protein levels in isolated satellite cells from indicated conditions. Tubulin served as a loading control. Full-length blots are presented in Supplementary Fig. S3. (**b**) Quantification of western blot results followed by ANOVA analysis shows the significant effect of Rbm24 knockdown. Rbm24 protein level in untransfected satellite cells is set to 1 as a reference, after normalization to tubulin. Data are the mean ± s.e.m. from three independent experiments. ANOVA F (df 2.6) = 6.182, *p* < 0.05; *p < 0.05 by Tukey’s HSD test. (**c**–**e**) Co-transfection of pEGFP-N1 and pCS2-RFP plasmids mixed at a 1:10 ratio. DAPI was used to stain nuclei. Note that all GFP-positive cells also expressed RFP. From this result, it can be extrapolated that, when pEGFP-N1 and shScramble or shRbm24 plasmids are used at the same ratio, GFP-labeled cells along with a proportion of unlabeled cells should be targeted by shRbm24. (**f**) Quantification of GFP-positive or RFP-positive cells. Data are the mean ± s.e.m. from three independent experiments. *p < 0.05 by Tukey’s HSD test. (**g**) Quantification of the overlapping expression of GFP with RFP in transfected satellite cells. Data are the mean ± s.e.m. from three independent experiments.

The increased expression of Rbm24 in newly formed myofibers during muscle regeneration raises the possibility that it may be involved in the differentiation of muscle stem cells in vivo. We sought to address this question by examining the differentiation capacity of Rbm24-silenced satellite cells transplanted to CTX-injured muscle. For this purpose, we generated a pRetro-SUPER-shRbm24 (shRbm24) plasmid, allowing the expression of a double stranded short hairpin RNA (shRNA) targeting *Rbm24* mRNA. A pRetro-SUPER-shScramble (shScramble) plasmid, which expresses a control shRNA that is not expected to target any mRNA, was used as a control. The efficiency and specificity of the shRbm24 were first examined by western blot analysis. Isolated satellite cells were transfected either with shScramble or with shRbm24. Compared to untransfected cells or shScramble-transfected cells, the level of Rbm24 protein was strongly reduced in shRbm24-transfected cells (Fig. 4a,b). This result ensures the efficiency and specificity of the shRbm24 for further experiments to silence Rbm24 expression.

In addition, to follow transplanted satellite cells in the regenerating muscle and to ensure that they are targeted by shRNAs, we first optimized the experimental conditions by transfecting cultured satellite cells with a mixture of pEGFP-N1 and pCS2-RFP plasmids at a 1:10 ratio. We reasoned that in this situation, all GFP-positive cells should also express RFP due to the large excess of the pCS2-RFP plasmid. By extrapolation, when satellite cells are transfected with pEGFP-N1 and shRbm24 plasmids at this ratio, all GFP-positive cells should be targeted by the shRNA, thus allowing the visualization of Rbm24-silenced satellite cells. Indeed, examination of fluorescence labeling indicated that there were generally twice more RFP-positive cells than GFP-positive ones, corresponding to a statistically significant difference (Fig. 4c–f). Most importantly, all GFP-positive cells also expressed RFP (Fig. 4g), indicating the suitability of this experimental design. Thus, it can be reasonably expected that not only all GFP-positive cells but also a proportion of unlabeled cells should contain shScramble or shRbm24 plasmid under these conditions.

### Rbm24 is involved in myogenic differentiation during muscle regeneration

**Figure 5:**
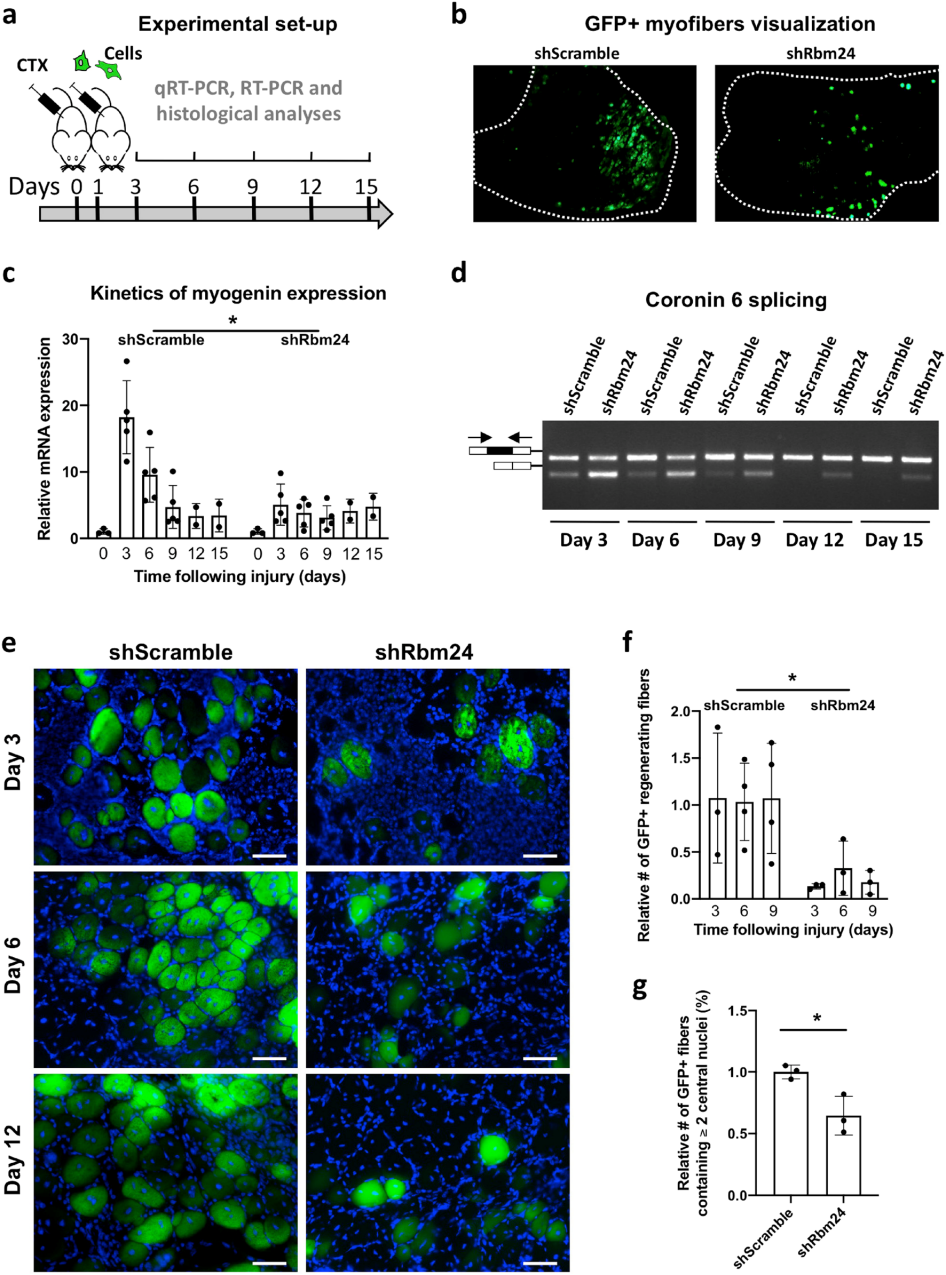
Rbm24 silencing affects skeletal muscle regeneration. (**a**) Muscle injury was induced by CTX injection in the tibialis anterior. Satellite cells previously transfected with GFP reporter and shScramble or shRbm24 at a 1:10 ratio were implanted in the injured area one day after injury. Regenerating muscles were collected at indicated time points. (**b**) Images illustrating transplanted satellite cells in the tibialis anterior 3 days after injury. (**c**) Analysis by qRT-PCR of *myogenin* mRNA expression. Rbm24 knockdown affects the increase of *myogenin* mRNA expression in regenerating muscle tissues 3 and 6 days after injury. Data are the mean ± s.e.m. from three independent experiments. Two-way ANOVA shows a significant effect of both variables, i.e. time and shRbm24, in the presence of a significant interaction. ANOVA for treatment F (df 1.8) = 8.370; time F (1.808, 8.679) = 11.41; interaction F (5.24) = 6.108, *p* < 0.05; *p < 0.05 by Tukey’s HSD test. (**d**) RT-PCR analysis of *coro6* muscle-specific alternative splicing. Rbm24 knockdown affects the inclusion of muscle-specific exon (black box) throughout the regeneration period. Full-length gel is presented in Supplementary Fig. S4. (**e**) Representative images of GFP-positive newly formed myofibers. Equal numbers of satellite cells (2 × 10^5^) were transplanted into CTX-injured tibialis anterior muscles. At different time points after injury, satellite cells transfected with shRbm24 produce less newly formed myofibers, compared to the control. DAPI was used to stain nuclei. Scale bar: 20 µm. (**f**) Quantification of newly formed myofibers at indicated time points after muscle injury. The number of GFP-positive cells in shScramble-transfected conditions is set to 1 as a reference. Data are the mean ± s.e.m. from three independent experiments. Two-way ANOVA shows a significant effect of shRbm24 independently of time. ANOVA for treatment F (df 1.14) = 18.28; *p < 0.05 by Tukey’s HSD test. (**g**) Quantification of GFP-positive newly formed myofibers containing at least two centralized nuclei at 6 days of regeneration. More than 100 regenerating myofibers were analyzed in each condition. Data are the mean ± s.e.m. from three independent experiments. *p < 0.05 by Student’s *t* test.

To examine whether and how Rbm24 is involved in skeletal muscle regeneration, freshly isolated satellite cells transfected with the pEGFP-N1 plasmid along with an excess of the shRbm24 plasmid were injected in the tibialis anterior of host mice the day after CTX injection (day 1). In each experiment, satellite cells transfected with the shScramble plasmid were injected in the contralateral tibialis anterior muscle injured by CTX. Regenerating muscles were harvested and analyzed at different time points (Fig. 5a). The integration of GFP-labeled satellite cells in the damaged muscle tissue was confirmed on cryosections 3 days after transplantation (Fig. 5b).

We first analyzed the temporal expression of known Rbm24 target genes in myoblasts and myofibers. Regenerating muscle tissue samples formed by transplanted satellite cells were enriched by manually dissecting GFP-positive regions. It has been reported that Rbm24 directly binds to and stabilizes *myogenin* mRNA to trigger early myogenic differentiation in vitro^31^, thus the temporal expression of *myogenin* mRNA was monitored by qRT-PCR analysis during regeneration. The results showed that *myogenin* mRNA level in the regenerating muscle tissue engrafted with control satellite cells (transfected with the shScramble plasmid) was strongly upregulated at early stages of regeneration, with more than a 15-fold increase at 3 days of regeneration and a nine-fold increase at 6 days. It was then decreased and remained constant at late stages of regeneration. In sharp contrast, when satellite cells were transfected with the shRbm24 plasmid, the upregulation of *myogenin* mRNA was severely impaired, with only a less than five-fold increase at 3 days of regeneration (Fig. 5c). These results demonstrate a requirement for Rbm24 in sustaining an elevated level of *myogenin* expression at early stages of muscle regeneration, which triggers the differentiation of satellite cells into new regenerating myofibers. The sharp increase in *myogenin* expression level at 3 days of regeneration is likely a consequence of its mRNA stabilization by Rbm24 in the cytoplasm of newly formed myofibers at more early stages. In addition, the constant and similar *myogenin* mRNA levels in control and Rbm24-silenced conditions after 6 days of regeneration imply that Rbm24 functions only at early stages to stabilize *myogenin* mRNA.

Rbm24 regulates a large number of muscle-specific alternative splicing events related to muscle differentiation and function^21^. We chose *coronin 6* (*coro6*) to examine its Rbm24-dependent inclusion of muscle-specific exon during regeneration, since it was shown to display progressive muscle-specific alternative splicing during myogenic differentiation in murine satellite cells^29^. RT-PCR analysis indicated that knockdown of Rbm24 affected the inclusion of muscle-specific exon in *coronin 6* pre-mRNA from 3 days until at least 15 days of regeneration (Fig. 5d). The requirement of Rbm24 for muscle-specific alternative splicing is consistent with its nuclear localization in newly formed myofibers at these stages. Altogether, the analyses of temporal gene expression suggest that Rbm24 is required for maintaining the stability of *myogenin* mRNA implicated in the early steps of myogenic differentiation, but for regulating alternative splicing of those mRNAs related to late steps of differentiation and muscular function.

Foreseeing that the Rbm24 loss of function would impact the regenerative process, we analyzed the effect of Rbm24 knockdown on myofiber formation following CTX-induced injury by examining the differentiation of GFP-positive myofibers on cryosections of regenerating muscles. Equal numbers (2 × 10^5^) of Rbm24-silenced or control cells were transplanted to the CTX-injured tibialis anterior muscle. At 3, 6, and 12 days of regeneration, we reproducibly observed that the number of GFP-positive myofibers in the injured muscle injected with Rbm24-silenced satellite cells was 2–2.5 times lower than that in the contralateral injured muscle injected with satellite cells transfected with the shScramble (Fig. 5e,f). The reduced number of GFP-positive myofibers caused by Rbm24 knockdown may be due to the defective differentiation and fusion of myoblasts. Indeed, the proportion of GFP-positive myofibers containing two or more centralized myonuclei was significantly reduced in the injured muscle injected with Rbm24-silenced satellite cells compared with the control muscle (Fig. 5g). These results suggest a reduced fusion index of satellite cells in the absence of Rbm24. This is correlated with the impaired upregulation of myogenin, which was implicated recently as an important regulator of muscle fusion^40,41^. Thus, the present results show that Rbm24 is involved in the differentiation of satellite cells into myofibers to repair the damaged tissue. This conclusion is consistent with the observation that Rbm24-silenced somitic myoblasts failed to differentiate into myofibers during embryogenesis^28^.

## Discussion

The present study identified a novel role of Rbm24 in skeletal muscle physiology. We showed that Rbm24 plays a role in the formation of myofibers during skeletal muscle regeneration, potentially through a fine-tuned regulation of multiple post-transcriptional events in differentiating satellite cells. We found that loss of Rbm24 function affects *myogenin* mRNA level in nascent myofibers at early stages of muscle regeneration and prevents muscle-specific alternative splicing during late stages of regeneration. As a result, myoblast fusion and differentiation into myofibers are impaired, as demonstrated by transplantation of satellite cells into CTX-injured muscle. These multiple functions of Rbm24 correlate with its dynamic cytoplasm to nucleus translocation during myogenic differentiation. Overall, our results suggest that Rbm24 represents a critical regulator that coordinates different aspects of post-transcriptional gene expression in regenerating muscles.

Rbm24 accumulates in the cytoplasm of fate-committed myoblasts and is involved in myogenic differentiation during embryonic development^28^, but whether it plays a role in adult muscle is not clear. We found the Rbm24 protein is exclusively localized to the myonuclei of type I and type IIa myofibers of the slow soleus muscle, as well as other fiber types in fast-twitch muscles such as gastrocnemius and tibialis anterior. There seems to be a higher level of Rbm24 expression in the slow muscle, but the functional implication needs further investigation. Using the more accessible tibialis anterior as a muscle injury model, we demonstrated that Rbm24 displays multiple functions during the course of regeneration. The requirement of Rbm24 for *myogenin* expression at early stages of muscle regeneration is consistent with an in vitro study demonstrating that Rbm24 binds to *myogenin* mRNA and regulates its stability in C2C12 cells^31^. It also coincides with the linear relationship in the expression of *Rbm24* and *myogenin* in different adult muscles. Moreover, it was shown recently that myogenin functions as an important regulator of muscle fusion^39^. Therefore, the reduced fusion index in Rbm24-silenced myofibers may be a consequence of impaired upregulation of *myogenin* expression at early stages of muscle regeneration.

How *myogenin* expression is controlled in myoblasts and particularly in activated satellite cells to trigger their fusion into myofibers is a topic of interesting research^40,41^. Many proteins have been shown to regulate the timing and cell-type specific transcription of *myogenin* gene^42–44^, but the molecular mechanism governing its post-transcriptional regulation is still poorly understood. The RBP HuR and the conserved long non-coding RNA lncMGPF have been identified as regulators of *myogenin* mRNA stability in regenerating muscles^45,46^. Although the regulation of *myogenin* mRNA stability by Rbm24 during myogenesis in vivo has not been determined so far, our present study clearly revealed an Rbm24-dependent increase of *myogenin* expression and myofiber differentiation during muscle regeneration, a process that largely recapitulates embryonic skeletal muscle development^6^. Nevertheless, the requirement of Rbm24 for *myogenin* expression may not fully account for its importance for the differentiation of newly formed fibers from satellite cells. It was reported that satellite cells from mice with muscle-specific conditional deletion of *myogenin* gene were able to proliferate and differentiate normally ex vivo^47^, whereas our results showed that Rbm24-silenced satellite cells fail to form myofibers in vivo, suggesting that Rbm24 plays multiple essential functions involved in muscle regeneration.

It has been well established that Rbm24 functions as a major regulator of muscle-specific alternative splicing by promoting the inclusion of muscle-specific exons involved in sarcomerogenesis^21^. While this work was ongoing, others were also tackling the same problem of Rbm24 function in muscle regeneration. Using a conditional knockout strategy, it was reported that Rbm24 regulates alternative splicing of genes involved in myofiber differentiation and myoblast fusion^48^. Interestingly, we found that *Rbm24* knockdown first inhibited *myogenin* expression and then prevented muscle-specific alternative splicing, indicating a temporally regulated post-transcriptional activity of this RBP during muscle regeneration. Thus, our present work both confirms and extends previous findings on the functional roles of Rbm24 in muscle physiology, with a temporally regulated manner.

These distinct functions of Rbm24 in RNA biogenesis are consistent with its dynamic cytoplasm to nucleus translocation during myogenic differentiation. Importantly, we made the first demonstration that Rbm24 protein is mostly distributed in the cytoplasm of myoblasts prior to fusion into nascent myotubes and then restricted to the myonucleus in differentiated myotubes and in adult muscles. The cytoplasmic localization of Rbm24 is fully compatible with its function in the stabilization of mRNAs encoding proteins involved in cell cycle arrest and fusion of myoblasts, such as p21 and myogenin^31,37^. There is now accumulating evidence that Rbm24 performs post-transcriptional functions in the cytoplasmic compartment of different cell types entered into the differentiation program. Indeed, our recent studies showed that both mouse and zebrafish Rbm24 is localized in the cytoplasm of differentiating cells in the head sensory organs to regulate mRNA stability and cytoplasmic polyadenylation^32,49,50^. This raises an interesting possibility that Rbm24 may also regulate the poly(A) tail length and thus the translational efficiency of muscle-specific mRNAs during early stages of muscle development and regeneration. As myogenic differentiation proceeds, the localization of Rbm24 in the myonucleus of myofibers coincides well with its function as a splicing factor that promotes the inclusion of muscle-specific exons from different genes involved in early differentiation, such as Mef2d^48^, or in the organization of sarcomere and the formation of neuromuscular junction^21,29^.

The implication of Rbm24 in several post-transcriptional events that take place in different cellular compartments raises the question of how its subcellular localization and function are regulated during muscle development and regeneration. This is of physiopathologically importance because it has been shown that mutations affecting the nuclear localization of RBPs, such as Rbm20, cause dilated cardiomyopathy^51,52^. Further studies by live cell imaging combined with the identification of its interacting partners and the analysis of post-translational modification should provide insights into the mechanism underlying this functionality-linked shuttling of Rbm24 between different subcellular compartments. In this regard, it has been shown that phosphorylation of Rbm24 could play a role in regulating its activity in mRNA translation^53^ and in modulating its stability in splicing-mediated sarcomere assembly^54^. In addition, Rbm24 interacts with several components of the cytoplasmic polyadenylation complex and regulates the poly(A) tail length and translational efficiency of lens-specific mRNAs^49^. Therefore, Rbm24 may perform context-dependent functions through interaction with its partners.

Another intriguing question also remains open. Our present study demonstrated that Rbm24 protein is specifically accumulated in the myonucleus of adult myofibers but not expressed in satellite cells. Thus, it could participate in several processes that maintain muscle homeostasis. On the one hand, its localization in the myonucleus clearly plays a role in regulating the splicing of muscle-specific structural and functional genes. On the other hand, its requirement for the differentiation of muscle stem cells into myofibers suggests an important role in promoting muscle regeneration. Thus, it will be of interest to understand when and how *Rbm24* gene becomes activated in these stem cells in response to muscle injury.

In summary, this work highlights a dynamic function of Rbm24 in governing myogenic differentiation during the muscle repair process. Our analyses on the localization and function of Rbm24 suggest that, all along its shuttling from the cytoplasm to the nucleus, it progressively converts undetermined myoblasts into differentiated myocytes, then promotes their fusion into myotubes, and finally organizes them into functional muscle fibers. During this process, Rbm24 plays key roles in several aspects of post-transcriptional regulation of muscle-specific gene expression, from mRNA stability to alternative splicing. Thus, our findings identify Rbm24 as a multifaceted regulator that coordinates myoblast differentiation during muscle development and regeneration.

## Methods

### Ethical statement

All experiments using animals were approved by the local Charles Darwin ethics committee n°5 in Paris and by the French Ministry of Higher Education and Research (project #1944). Mice were treated strictly according to the guidelines of the Institutional Animal Care and Use Committee and to relevant national and European legislation (Directive 2010/63/EU). Authors declare that all animal care and experimental procedures were performed in accordance with the ARRIVE guidelines.

### Expression constructs

Rbm24, Rbm24∆RRM and Rbm24RRM coding sequences were cloned in the pEGFP-C1 vector (Clontech) to generate the corresponding expression constructs. Silencing of Rbm24 was achieved by designing a short hairpin targeting the mouse Rbm24 sequence (5′-AGCTGCTGCAGGCTATGTAACC-3′*).* An shScramble sequence (5′-CCTAAGGTTAAGTCGCCCTCG-3′) was used as a control shRNA. A 52-bp double-stranded oligonucleotide consisting of a palindromic loop sequence (sense-TTCAAGAGA-antisense), flanked by HindIII and BglII restriction sites, was cloned into the pRetro-SUPER vector downstream of the H1 RNA polymerase III promoter to generate pRetro-SUPER-shRbm24 and pRetro-SUPER-shScramble constructs. All cloned sequences were verified by Sanger DNA sequencing (GATC Biotech).

### RNA extraction and RT-PCR analyses

Skeletal muscle tissues were manually dissected from adult mice that were euthanized by cervical dislocation. Total RNA was extracted using TRIzol Reagent (Invitrogen), according to the manufacturer’s protocol. One microgram of total RNA was reverse transcribed using M-MLV reverse transcriptase (Invitrogen) in the presence of random primers. The analysis of gene expression by qPCR was performed in a CFX96 real-time PCR detection system using SsoFast EvaGreen SuperMix (Biorad) with gene-specific primers (*myogenin*: 5′-CAATGCACTGGAGTTCG-3′ and 5′-ACGATGGACGTAAGGGAGTG-3′; *Rbm24*: 5′-CTTGGGAGCAAAACCAAGA-3′ and 5′-GAAGCTGTTGAACGCCAAA-3). *Glyceraldehyde-3-phosphate dehydrogenase* (*GAPDH*) was used as an input control (5′-CCTCTGACTTCAACAGCGAC-3′ and 5′-CGTTGTCATACCAGGAAATGAG-3′). The analysis of *coronin 6* alternative splicing isoform by RT-PCR was done using specific primers as described^29^. All experiments were performed using three to five independent samples analyzed in duplicate.

### Culture and transfection of C2C12 cells

Mouse C2C12 myoblasts (ATCC) were cultured on 6-well plates and grown in Dulbecco's modified Eagle's medium (DMEM) with 10% (v/v) fetal bovine serum (FBS), l-glutamine (4 mM), glucose (4.5 g/L), penicillin (100 units/mL), and streptomycin (100 μg/mL). They were transiently transfected at 80% confluence with plasmid DNA (4 µg) using lipofectamine 2000 reagent (ThermoFisher Scientific), according to the manufacturer’s instructions, and were induced to undergo differentiation by replacing 10% FBS with 2% horse serum in the culture medium^55^.

### Isolation and transfection of satellite cells

Satellite cells were isolated according to published protocol^56^. Mouse hindlimb muscles (tibialis anterior, extensor digitorum longus, quadriceps, gastrocnemius, soleus) and diaphragm were harvested from one adult, freed of facias and tendons, and cut into small pieces in drops of pre-warmed serum-free DMEM. Muscle pieces were digested in 0.1% pronase solution (Sigma-Aldrich) for 1 h at 37 °C on an orbital shaker. Dissociated cells were collected by centrifugation at 400*g* for 5 min and resuspended in pre-warmed DMEM containing 10% horse serum. Satellite cells were released from the bulk of cells by successive mechanical trituration using 10 mL and 5 mL serological pipettes. The supernatant with released cells was filtered through a 40 µm cell strainer, and the single cell suspension was centrifuged at 400*g* for 10 min. The cell pellet was resuspended in 10 mL of DMEM containing FBS (20%), chicken embryo extract (1%), and penicillin/streptomycin (1%), plated on a 10 cm Petri dish and cultured for 90 min in an incubator at 37 °C with CO_2_ supply (5%). Because fibroblasts adhere to the Petri dish, satellite cells floating in the medium were transferred into Matrigel-coated Petri dishes at a density of 1 × 10^5^ cells/cm^2^ and cultured for 24 h before transfection using Lipofecatime 3000 reagent (ThermoFisher Scientific). An optimal ratio of DNA/Lipofectamine 3000 at 1:3 provided the highest transfection efficiency up to 30%. In co-transfection experiments, the GFP reporter and shRNA plasmid were mixed at a ratio of 1:10 to allow all GFP-positive cells concomitantly expressing the shRNA.

### Muscle injury and transplantation of satellite cells

RjOrl:SWIISS female mice at 8–10 weeks of age were used in the study. Left tibialis anterior muscles were damaged by intramuscular injection of 50 µL of 10 µM CTX (Sigma-Aldrich) dissolved in PBS, as described^57^. Right tibialis anterior muscle injected with PBS was used as control. Cell transplantation was performed by injecting 2 × 10^5^ cells suspended in 50 µL of DMEM into the damaged muscles at day 1 after injury. At 3, 6, 9, 12 and 15 days after cell transplantation, mice were euthanized, and GFP-positive regions were manually dissected under a fluorescent microscope.

### Immunofluorescence staining

Muscle tissues were embedded in tissue-freezing medium (Leica, Germany) and frozen in liquid nitrogen-cooled isopentane. Transverse cryosections at 14 μm were obtained using a microtome cryostat (Leica, Germany) and fixed in 4% paraformaldehyde for 10 min at room temperature. For immunofluorescence staining, sections were permeabilized with Triton-X100 (0.2%) in PBS for 30 min, blocked in saturation solution (3% bovine serum albumin, 0.1% Triton-X100 in PBS) for 1 h, and incubated overnight at 4 °C with primary antibodies diluted in the saturation solution (rabbit polyclonal antibody against Rbm24 from Proteintech, 1:500; clone BA-F8 MYH7 monoclonal antibody against MyHC-I from Developmental Studies Hybridoma Bank, 1/40; clone SC-71 MYH2 monoclonal antibody against MyHC-IIa from Developmental Studies Hybridoma Bank, 1/200; monoclonal antibody against dystrophin from Novocastra, 1:200; rabbit polyclonal antibody against laminin from abcam, 1:100; clone F1.652 monoclonal antibody against eMHC from Developmental Studies Hybridoma Bank, 1:20). After three washes in PBT (0.1% Tween‐20 in PBS), sections were incubated for 1 h in alexa‐488 or alexa-596 conjugated anti‐rabbit (1:400) or anti‐mouse (1:400) secondary antibodies (Interchim), washed in PBT, and counterstained with DAPI before mounting in Dako Fluorescent Mounting Medium. C2C12 myoblasts and myotubes expressing GFP fusion proteins were also fixed in 4% paraformaldehyde for 10 min at room temperature, and counterstained with DAPI. Images were taken using a Zeiss Axioimager apotome.

### Western blot analysis

Muscle tissues and satellite cells were homogenized in lysis buffer (50 mM Tris–HCl, pH 7.4, 1 mM EDTA, 150 mM NaCl, 1% Triton-X100) supplemented with cOmplete, Mini, EDTA-free Protease Inhibitor Cocktail and PhosSTOP Phosphatase Inhibitor Cocktail (both from Sigma-Aldrich). Protein samples were separated by SDS-PAGE and transferred to nitrocellulose sheets (GE Healthcare). Unspecific bindings were blocked using non-fat dry milk (5%) in TBST (20 mM Tris–HCl, pH 7.5, 137 mM NaCl, 0.1% Tween-20) for 1 h at room temperature. The nitrocellulose membranes were then incubated at 4 °C overnight with Rbm24 antibody (abcam, 1:200) or α-tubulin antibody (Sigma-Aldrich, 1:10,000) diluted in TBST containing bovine serum albumin (5%). After washing in TBST, the membranes were incubated for 1 h at room temperature with horseradish peroxidase-conjugated anti-mouse or anti-rabbit secondary antibodies (Biorad), and protein bands were visualized using the Western Lighting Plus-ECL kit (PerkinElmer). The intensity of signals was quantified using ImageJ software (version number 1.45s).

### Statistical analysis

Comparisons of quantitative variables were performed through 1-way or 2-way ANOVA, after verifying parametric assumptions. In case these assumptions were violated, a transformation (square root) was used. Post hoc comparisons were performed through Tukey’s or Student’s *t* test. The significance level was set at *p* < 0.05. Statistical analyses were performed by Prism software (version number 9.0.1).

## Supplementary Information

Supplementary Figures.
