# Supplementary material for: Rbm24 displays dynamic functions required for myogenic differentiation during muscle regeneration
Source: Sci Rep. 2021 May 3;11:9423. doi: 10.1038/s41598-021-88563-3 (PMC8093301; doi:10.1038/s41598-021-88563-3)
Supplement: Supplementary file 1 — Supplementary Figures. [file 41598_2021_88563_MOESM1_ESM.pdf]

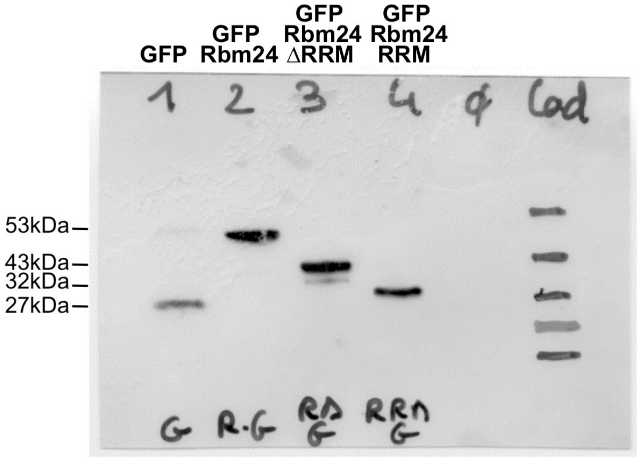

Supplementary Figure S1. Crude image of uncropped scan of the western blot corresponding to Figure 2b in the main text and representing the analysis of fusion proteins indicated in figure 2a in C2C12 myoblasts 24 h after transfection using a GFP antibody.

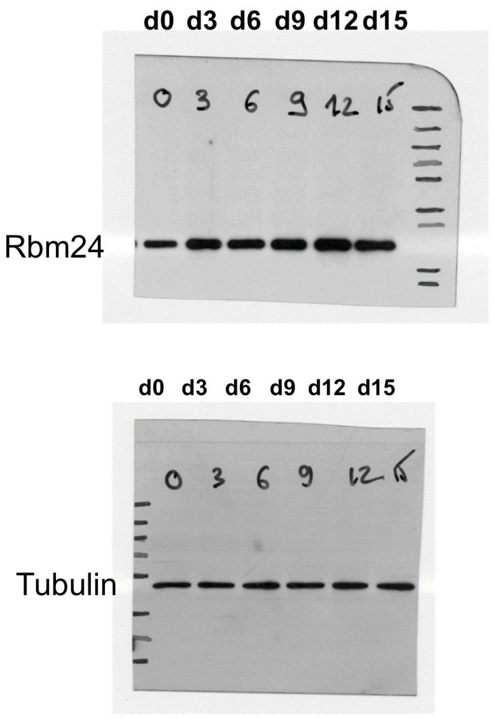

Supplementary Figure S2. Crude image of uncropped scan of the western blot corresponding to Figure 3a in the main text and representing the analysis of Rbm24 protein in the tibialis anterior muscle of adult mice at 3, 6, 9, 12 and 15 days (d) of regeneration. Control muscle was the contralateral tibialis anterior injected with PBS. Tubulin was used as a loading control.

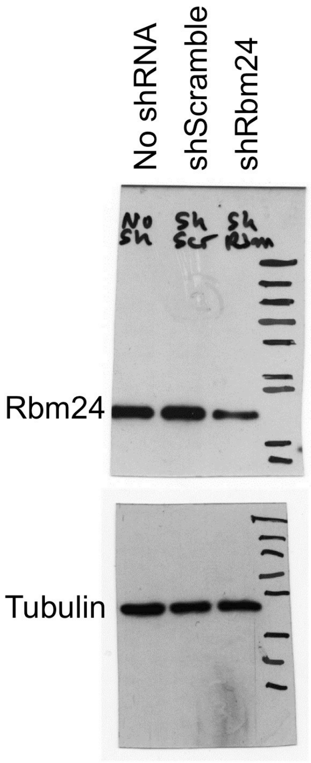

Supplementary Figure S3. Crude image of uncropped scan of the western blot corresponding to Figure 4a in the main text and representing the analysis of Rbm24 protein levels in isolated satellite cells from indicated conditions. Tubulin served as a loading control.

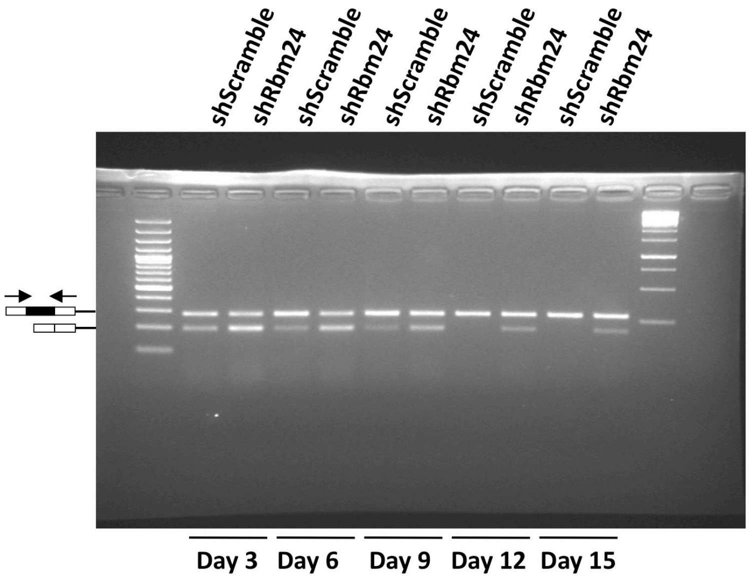

Supplementary Figure S4. Crude image of uncropped scan of PCR products in agarose gel corresponding to Figure 5d in the main text and representing the RT-PCR analysis of muscle-specific alternative splicing of *coro6* gene in regenerating muscle following Rbm24 knockdown. Rbm24 knockdown affects the inclusion of muscle-specific exon (black box) throughout the regeneration period.
